# The cellular stress sensor HSPB1 regulates the membrane localization of amino acid transporter SLC7A5 in breast cancer

**DOI:** 10.1016/j.jbc.2026.113197

**Published:** 2026-05-27

**Authors:** Yukako Suzuki, Narumi Kitayama, Ryuhei Kudo, Karishma Silwal, Shiori Matsuda, Maki Ohishi, Ayano Ueno, Sanae Ashitani, Kaori Igarashi, Takeshi Masuda, Takamasa Ishikawa, Tomoyoshi Soga, Yasuhiro Saito

**Affiliations:** 1 Institute for Advanced Biosciences, Keio University, Tsuruoka, Yamagata, Japan; 2 Systems Biology Program, Graduate School of Media and Governance, Keio University, Fujisawa, Kanagawa, Japan; 3 Infinity Lab Co., Ltd, Tsuruoka, Yamagata, Japan; 4 Human Biology-Microbiome-Quantum Research Center (WPI-Bio2Q), Keio University, Tokyo, Japan

**Keywords:** heat shock protein, amino acid transport, breast cancer, drug resistance, polarity protein

## Abstract

The heat shock protein beta-1 (HSPB1/HSP27) is highly expressed and phosphorylated in cancer tissues. However, the precise role of HSPB1 in cancer remains unclear. In this study, we report the unexpected findings elucidating the essential role of HSPB1 in adapting amino acid deficiency by upregulating amino acid transporter SLC7A5 function. HSPB1 regulates estrogen receptor-positive (ER+) breast cancer cell proliferation in a SLC7A5-dependent manner. In response to cellular stress, which is specified as amino acid-deficient conditions, HSPB1 was phosphorylated at Ser 78 residue by stress MAPK p38. SLC7A5 is associated with phosphorylated HSPB1 for its functional activation, leading to upregulated amino acid incorporation. In addition, HSPB1 and SLC7A5 overexpression increased acetylated α-tubulin levels. SLC7A5 overexpression did not change acetyl-CoA level, but SLC7A5 knockdown decreased ATAT1 and induced HDAC6 upregulation. Furthermore, HSPB1 and SLC7A5 induced paclitaxel and tamoxifen resistance. Therefore, the HSPB1-SLC7A5 axis contributes to the acquisition of tolerance to both tamoxifen and paclitaxel in breast cancer cells, uncovering a novel therapeutic target against drug resistance in breast cancer.

Breast cancer is one of the leading causes of death worldwide, with approximately 75% cases driven by aberrant expression of estrogen receptor (ER) (1, 2). Although patients with ER+ disease are eligible for endocrine therapy, the development of resistance and metastatic progression remains a paramount challenge in patients with breast cancer. Thus, identifying new vulnerabilities to combat resistance to anti-estrogen treatment is imperative in breast cancer research.

Understanding how cancer cells meet their high demand for nutrients and essential amino acids remains a poorly understood aspect. They are thought to adapt by increasing nutrient and amino acid uptake or degrading and recycling cellular contents *via* autophagy (3). Although the precise mechanisms through which cancer cells adapt to nutrient stress and modulate essential amino acid uptake have not been fully elucidated, it is increasingly evident that they can sense limitations in the intracellular essential amino acid concentration using an evolutionarily conserved mechanism involving mechanistic target of rapamycin complex 1 (mTORC1) and general control non-derepressible 2 (GCN2) (4, 5). The molecular mechanisms of amino acid sensing and the autophagy activation by mTORC1 are extensively investigated, but how amino acid uptake is regulated in response to nutrient stress is still unclear.

The L-type amino acid transporter SLC7A5/LAT1 is a bidirectional amino acid transporter that incorporates essential amino acids into cells, and the aberrant expression is broadly observed in many tumor tissues, such as breast, lung, esophageal, and kidney cancers (6, 7, 8, 9). Therefore, SLC7A5 is considered a promising target for anticancer drugs (10, 11). Besides, SLC7A5 is a primary amino acid transporter that uptakes essential amino acids in growth-limited conditions (12), suggesting that its functions should be adjusted depending on environmental nutrient conditions. However, the precise regulatory mechanisms of functional SLC7A5 upregulation are poorly understood.

Our previous report showed a biological mechanism whereby the cell polarity proteins LLGL2 and SCRIB regulate membrane localization of SLC7A5 and thereby facilitate leucine uptake (13, 14). This heightened SLC7A5 activity in ER+ breast cancer cells leads to aberrant cell proliferation and tamoxifen drug resistance, providing a novel therapeutic opportunity to combat endocrine therapy-resistant breast cancer cells. Furthermore, we have demonstrated that the LLGL2-SLC7A5 interaction is enhanced by nutrient-limited conditions called nutrient stress in ER+ breast cancer cells. This suggests that the SLC7A5 function is regulated by complex formation with LLGL2 in response to intracellular or extracellular nutrient levels. However, the molecular mechanisms underlying the nutrient-sensing mechanisms of the LLGL2-SLC7A5 complex remain unclear.

Heat shock proteins (HSPs) are chaperones that prevent protein aggregation and help refold denatured or misfolded proteins (15). HSPB1/HSP27 is a major heat shock protein that is highly expressed in tumor tissues, including breast, bladder, colon, lung, ovarian, and prostate cancer tissues (16, 17, 18, 19, 20, 21, 22). The chaperone functions of HSPB1 in cancer cells have been extensively investigated. However, neither its role nor its mechanism of action in tumors are well-understood.

Here, we report unexpected HSPB1 functions as a cellular stress sensor including amino acid deficiency, to regulate SLC7A5 function in response to amino acid-depleted conditions. We discovered that HSPB1 interacts with SLC7A5, resulting in the dysregulation of amino acid metabolism. We showed that phosphorylated HSPB1 is indispensable for the LLGL2-SLC7A5 interaction. Therefore, phosphorylated HSPB1 functions as a cellular stress sensor under amino acid-depleted conditions for SLC7A5 activation in ER+ breast cancer cells. In addition, we uncovered that α-tubulin acetylation is regulated by the HSPB1-SLC7A5 pathway, thereby acquiring drug resistance against paclitaxel, a microtubule-targeting drug, in ER+ breast cancer cells. In addition to the finding that HSPB1 induced tamoxifen resistance, the HSPB1-SLC7A5 complex induces tolerance to both tamoxifen and paclitaxel in breast cancer cells. These data reveal a new therapeutic opportunity to counteract drug resistance in breast cancer.

## Results

### HSPB1 affects cell proliferation in ER+ breast cancer

**Figure 1:**
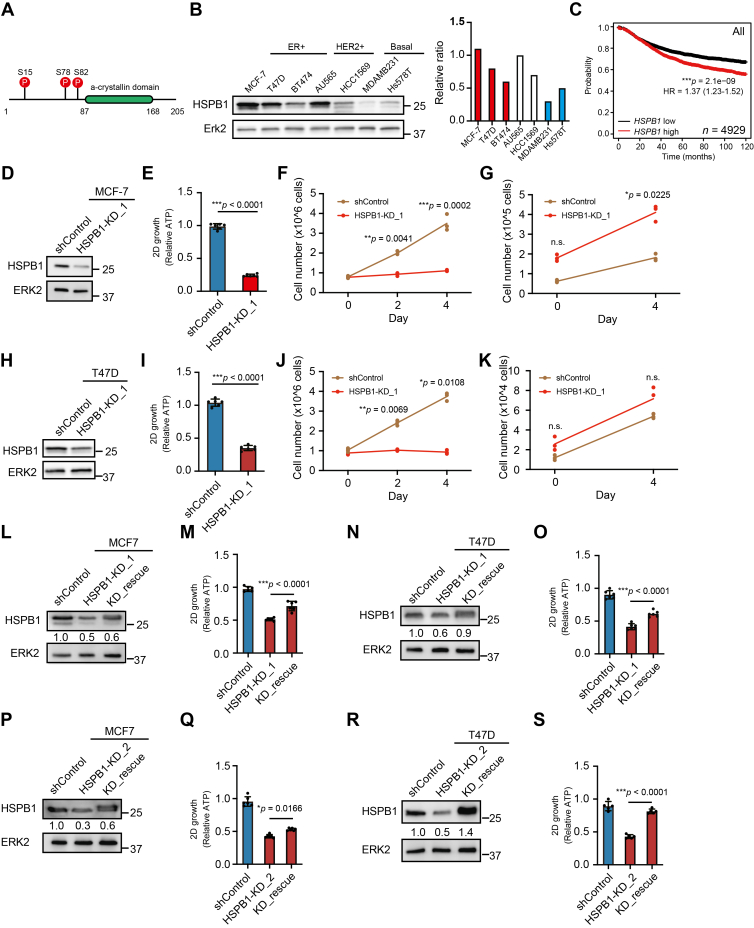
**HSPB1 regulates cell proliferation in ER+ breast cancer cells.***A*, cartoon representation of HSPB1. *B*, total protein levels of HSPB1 in breast cancer cell lines. Immunoblot images (*left*) and the relative ratio of signal intensity (*right*). *C*, Kaplan-Meier plot of breast cancer patients’ survival. *D*, Knockdown of HSPB1 (HSPB1-KD) in MCF-7 cells. *E*, relative cell number of HSPB1-KD MCF-7 cells by measuring ATP amount using Celltiter-glo 3D. *F*, The living cell number of HSPB1-KD MCF-7 cells. *G*, the dead cell number of HSPB1-KD MCF-7 cells. *H*, Knockdown of HSPB1 in T47D cells. *I*, relative cell number of HSPB1-KD T47D cells by measuring ATP amount by using Celltiter-glo 3D. *J*, the living cell number of HSPB1-KD T47D cells. *K*, the dead cell number of HSPB1-KD T47D cells. *L*, Immunoblot images of HSPB1-KD rescue in MCF7 cells. *M*, Relative cell number of HSPB1-KD and the HSPB1-KD rescued MCF-7 cells by measuring ATP amount by using Celltiter-glo 3D. *N*, Immunoblot images of HSPB1-KD rescue in T47D cells. *O*, relative cell number of HSPB1-KD and the HSPB1-KD rescued T47D cells by measuring ATP amount by using Celltiter-glo 3D. Data (*E*, *F*, *G*, *I*, *J*, *K*, *M*, *O*, *Q* and *S*) are shown as mean ± s.d.; *n* = 6 for relative ATP or n = 3 for cell counting. Statistical analysis was conducted by two-tailed *t* test (*E*, *I*), one-way ANOVA followed by Tukey’s post-test (*M*, *O*, *Q*, *S*), or two-way ANOVA followed by Sidak’s post-test (*F*, *G*, *J, K*).

The primary structure of HSPB1 is highly homologous to other members of the HSP family, which share the conserved α-crystallin domain (Fig. 1*A*) (23). HSPB1 also contains three serine residues in the N-terminal region (residues 15, 78, and 82). Both HSPB1 overexpression and highly phosphorylated HSPB1 are frequently observed in breast cancer tissues (19). Therefore, we examined HSPB1 expression in cultured breast cancer cells. HSPB1 was widely expressed in breast cancer cells, but was highly expressed in ER+ cell lines compared to that in ER– breast cancer cells (Fig. 1*B*). Furthermore, the analysis of *HSPB1* mRNA expression in patients with breast cancer (*n* = 4929) showed a correlation between elevated *HSPB1* mRNA levels and poor clinical prognosis (Fig. 1*C*). Furthermore, this association held true for both ER+ (*n* = 2633) and ER– (*n* = 1190) breast cancer cases (Fig. S1, *A* and *B*), prompting us to delve deeper into the pathological role of HSPB1 in breast cancer cells.

We explored the effect of HSPB1 on cell proliferation by generating HSPB1-knockdown (HSPB1-KD) MCF-7 cells using two short hairpin RNAs (shRNAs) targeting different sequences in the *HSPB1* gene. HSPB1-KD reduced cell number in MCF-7 cells (Fig. 1, *D* and *E*). HSPB1-KD increased dead cell number in MCF-7 cell (Figs. 1, *F*, *G* and S1, *C*, *D*). Meanwhile, HSPB1-KD reduced cell number in T47D cells (Fig. 1, *H* and *I*). In contrast to MCF-7 cells, HSPB1-KD did not change the dead cell number in T47D cells (Figs. *J*, *K* and S1, *E*, *F*). HSPB1-KD suppressed cell proliferation without changing dead cell number in BT474 cells (Fig. S1, *G*–*K*), indicating that HSPB1 positively regulates cell proliferation in breast cancer cells. The protein reduction of HSPB1 by shRNA was rescued by expressing shRNA-resistant HSPB1. In both MCF-7 and T47D cells, the growth defect by HSPB1-KD could be rescued (Fig. 1, *L*–*S*), indicating that the phenotypic rescue was achieved in ER+ breast cancer cells. In addition, HSPB1-KD reduced the cell number of ZR75-1, MDA-MB231, and Hs578T cells (Fig. S1, *L*–*Q*). Conversely, HSPB1-overexpressing (HSPB1-OE) increased cell number in MCF-7, T47D, and ZR75-1 cells, but not in MDA-MB-231, Hs578T, and BT549 cells, suggesting that HSPB1-OE positively regulates cell proliferation in only ER+ breast cancer cells (Fig. S1, *R*–*Y*).

### HSPB1 binds with SLC7A5 in ER+ breast cancer cells

**Figure 2:**
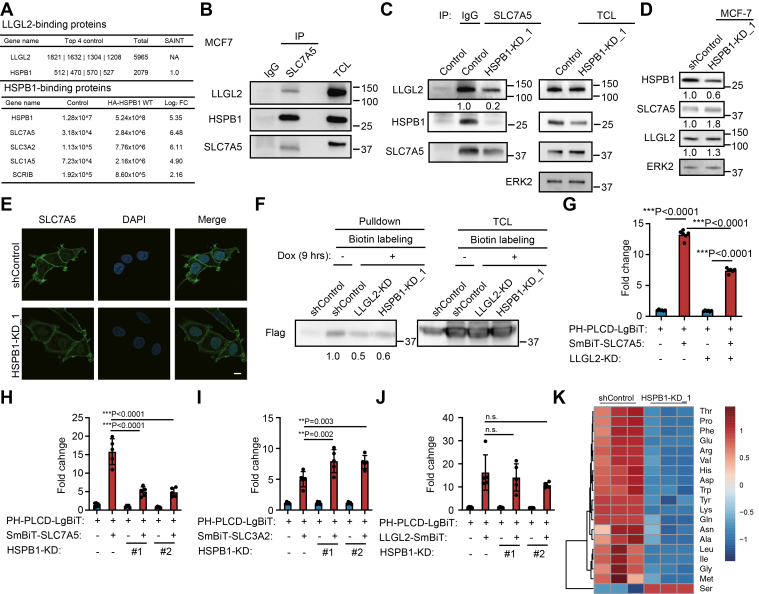
**HSPB1 is essential for the LLGL2-SLC7A5 complex formation.***A*, list of LLGL2-and HSPB1-binding proteins in ER+ breast cancer cells. LLGL2-binding proteins are shown as the number of peptides whereas HSPB1-binding proteins are shown as the relative area. *B*, endogenous HSPB1-LLGL2 and HSPB1-SLC7A5 interaction in MCF-7 cells. The immunoprecipitation is performed using anti-SLC7A5 antibody. *C*, endogenous LLGL2-SLC7A5 interaction in HSPB1-KD MCF-7 cells. The immunoprecipitation is performed using anti-SLC7A5 antibody. *D*, total protein levels of SLC7A5 and LLGL2 in HSPB1-KD MCF-7 cells. *E*, Immunostaining images for SLC7A5 (*green*) and DAPI (*blue*) in control (shControl) and HSPB1-KD MCF-7 cells. Scale bar indicates 10 μm. *F*, cell surface protein levels of SLC7A5 in LLGL2-KD or HSPB1-KD MCF-7 cells. *G*, the evaluation of the membrane-localized SLC7A5 in LLGL2-KD cells. *H*, The evaluation of the membrane-localized SLC7A5 in HSPB1-KD cells. *I*, the evaluation of the membrane-localized SLC3A2 in HSPB1-KD cells. *J*, the evaluation of the membrane-localized LLGL2 in HSPB1-KD cells. *K*, heatmap of down- or upregulated amino acids in HSPB1-KD MCF-7 cells.

In our previous paper (13), LLGL2 interactome data listed HSPB1 as a top LLGL2 interactant with a Significance Analysis of INTeractome (SAINT) score of 1.0 (24) (Fig. 2*A*). HSPB1 interacts with SLC7A11/xCT, which has a similar structure and complex formation with SLC7A5 (25, 26). Given their structural similarity, HSPB1 may have a function similar to that of SLC7A5 in ER+ breast cancer cells. However, the role of HSPB1 in ER+ breast cancer cells remains unclear. Thus, we explored HSPB1-interacting proteins to uncover the molecular mechanisms of HSPB1-dependent cell proliferation in ER+ breast cancer cells. We performed an interactome analysis using hemagglutinin (HA)-tagged HSPB1. After overexpressing HA-tagged HSPB1 in MCF-7 cells and the cell lysates were immunoprecipitated with anti-HA antibodies and analyzed using mass spectrometry. We identified 4908 proteins in the wild-type HSPB1 precipitates, including known HSPB1-binding proteins such as β-catenin, Glucose-6-phosphate 1-dehydrogenase (G6PD) (27, 28, 29) (Supplementary Tables S1 and S2). Notably, SLC proteins (SLC7A5, SLC3A2, and SLC1A5) and the polarity protein SCRIB were detected as potential HSPB1-interacting molecules (Fig. 2*A*). SLC7A5 is essential for cell proliferation and therapeutic resistance in ER+ breast cancer. SLC3A2 and the polarity proteins LLGL2 and SCRIB form a heteroquaternary complex with SLC7A5 (13, 14). Re-analyzing, supporting that HSPB1 targets SLC7A5 complex in ER+ breast cancer cells.

### HSPB1 is essential for LLGL2-SLC7A5 complex formation

To confirm the findings of the interactome analysis (Fig. 2*A*), we examined the HSPB1-LLGL2 interaction in MCF-7 cells using immunoprecipitation. Cell lysates from MCF-7 or T47D cells were immunoprecipitated with an anti-SLC7A5 antibody, and the precipitates were immunoblotted with the indicated antibodies. We observed SLC7A5-LLGL2 and SLC7A5-HSPB1 interactions at the endogenous protein level (Figs. 2*B* and S2*A*). In addition, we confirmed the complex formation by reciprocal immunoprecipitation using anti-HSPB1 antibody (Fig. S2, *B* and *C*). Next, we examined whether HSPB1 is a component of the LLGL2-SLC7A5 complex using immunoprecipitation. Notably, the LLGL2-SLC7A5 interaction was remarkably impaired in HSPB1-KD cells at endogenous protein level (Fig. 2*C*). SLC7A5 is colocalized with HSPB1 in MCF-7 cells (Fig. S2*D*). We also confirmed the SLC7A5 and HSPB1 colocalization by quantifying the signal intensity of immunostaining image (Fig. S2*E*). The peak of SLC7A5 and HSPB1 signals are overlapped at the same position, suggesting that SLC7A5 and HSPB1 colocalize at the plasma membrane (Fig. S2*E*). Furthermore, LLGL2-HSPB1 association requires the N-terminal region of LLGL2 (Fig. S2, *F* and *G*), a region crucial for its interaction with SLC7A5 (13). This indicates that the element facilitating LLGL2’s interaction with both HSPB1 and SLC7A5 is identical to that shown in our previous report (30). Furthermore, LLGL2-polybasic mutants that cannot translocate to the plasma membrane (LLGL2-Pb) (31) could interact with HSPB1 and SLC7A5, indicating that the LLGL2-HSPB1 complex is formed in the cytosol, akin to the LLGL2-SLC7A5 interaction (Fig. S2, *F*–*H*). These results suggest that HSPB1 is an essential component of the LLGL2-SLC7A5 complex in ER+ breast cancer cells.

### HSPB1 is required for the function of SLC7A5 in ER+ breast cancer cells

Our previous report showed the vital role of LLGL2-SLC7A5 interaction in shuttling SLC7A5 to the plasma membrane and the function of SLC7A5 in ER+ breast cancer cells (13). We investigated the effect of HSPB1-KD on the intracellular localization of SLC7A5 in ER+ breast cancer cells. HSPB1-KD in MCF-7 and T47D cells did not decrease the protein levels of SLC7A5 and LLGL2 (Figs. 2*D* and S2*I*). However, the membrane-localized SLC7A5 was dispersed by HSPB1-KD in these cells (Figs. 2*E* and S2, *J*, *K*). Surface protein levels of SLC7A5 were also downregulated by HSPB1-KD in MCF-7 cells (Figs. 2*F* and S2*K*). To validate the membrane localization of SLC7A5, we established Nano-Bit expression system. LgBiT was fused with PH domain of PLC delta and constitutively expressed in MCF-7 cells. Then, a tet-on inducible SmBiT-SLC7A5 was introduced into PH-LgBiT-expressing MCF-7 cells. At 16 h from the doxycycline addition, the signal intensity of luciferase was drastically increased (Fig. 2*G*). Meanwhile, when LLGL2 is downregulated, signal intensity is impaired (Fig. 2*G*), suggesting that this Nano-BiT system reflects the membrane localization of SLC7A5 in MCF-7 cells. HSPB1-KD also reduced luciferase activity in SmBiT-SLC7A5-induced MCF-7 cells (Fig. 2*H*), indicating that HSPB1 regulates the membrane localization of SLC7A5. We further developed the Nano-BiT system using SLC3A2 and LLGL2. HSPB1-KD slightly increased the membrane localization of SLC3A2, whereas HSPB1-KD did not affect the membrane localization of LLGL2 (Fig. 2, *I* and *J*). Consistent with the downregulation of SLC7A5 in the plasma membrane of HSPB1-KD cells, metabolome analysis revealed that HSPB1-KD led to a reduction in the intracellular amino acid levels, including leucine (Figs. 2*K* and S2*L*). Taken together, these results highlight the essential role of HSPB1 in LLGL2-SLC7A5 interaction and membrane localization of SLC7A5 in ER+ breast cancer cells.

### HSPB1 phosphorylation is required for SLC7A5 interaction

**Figure 3:**
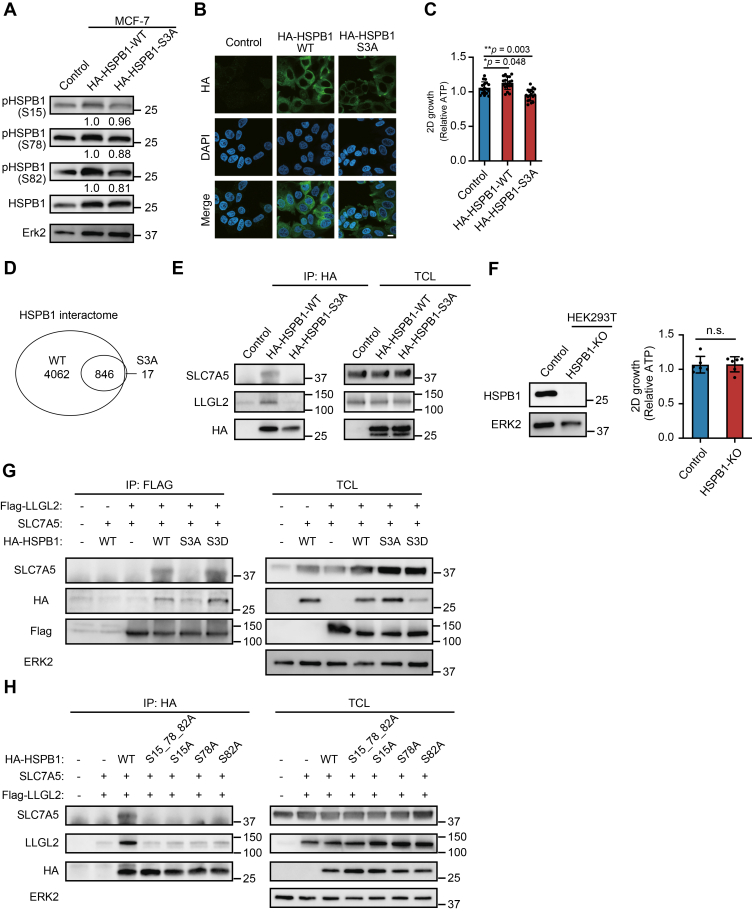
**HSPB1 phosphorylation is required for LLGL2-SLC7A5 interaction.***A*, phosphorylation levels of HA-tagged wild-type HSPB1 (HA-HSPB1-WT) and phosphorylation-resistant mutant of HSPB1 (HA-HSPB1-S3A) in wild-type MCF-7 cells. The signal intensity of phosphorylated HSPB1 was normalized with the intensity of total HSPB1. *B*, immunostaining images for HA-tagged HSPB1 (*green*) and DAPI (*blue*) in control, HA-HSPB1-WT-expressing, and HA-HSPB1-S3A-expressing MCF-7 cells. Scale bar indicates 10 μm. *C*, relative cell number of HA-HSPB1-WT or HA-HSPB1-S3A-expressing MCF-7 cells by measuring ATP amount by Celltiter-glo 3D. *D*, Venn diagram representation of the number of binding proteins with HA-HSPB1-WT and/or HA-HSPB1-S3A in MCF-7 cells. *E*, HA-HSPB1-WT or HA-HSPB1-S3A was expressed in wild-type MCF-7 cells, and the cell lysates were immunoprecipitated with anti-HA antibody. The precipitates were immunoblotted with the indicated antibodies. *F*, HSPB1-knockout (HSPB1-KO) HEK293T cells were prepared by CRISPR-Cas9 system. HSPB1-KO was confirmed by immunoblot (*left*). The 2D cell proliferation was assessed at day 3 and relative cell number was measured by using Celltiter-glo 3D (*right*). *G*, Flag-LLGL2, SLC7A5, and HA-HSPB1 are expressed in HSPB1-KO HEK293T cells and the cell lysates were immunoprecipitated with anti-Flag antibody. The precpitates were immunoblotted with the indicated antibodies. *H*, Flag-LLGL2, SLC7A5, and HA-HSPB1 mutants were expressed in HSPB1-KO HEK293T cells. The cell lysates were immunoprecipitated with anti-HA antibody and the precipitates were immunoblotted with the indicated antibodies. Data (*C*, *F*) are shown as mean ± s.d., *n* = 18 (*C*) n = 6 (*F*) from three independent experiments with six biological replicates. Statistical analysis was conducted by two-tailed *t* test (*F*) and one-way ANOVA followed by Tukey’s post-test (*C*).

HSPB1 is phosphorylated at Ser 15 (S15), Ser 78 (S78), and Ser 82 (S82). MAPK-activated protein (MAPKAP) kinase two-thirds, ribosomal protein S6 kinaseA1/p90Rsk, and cyclic guanosine monophosphate-dependent protein kinase (PKG) phosphorylate HSPB1 at S78 and S82, whereas protein kinase C (PKC) phosphorylates at S15 and S78. In addition, protein kinase D (PKD) phosphorylates HSPB1 at S82 (32, 33). The phosphorylation of the three serine residues has been associated with the dissociation of large HSPB1 oligomers, thereby enhancing its chaperone activity (34, 35). HSPB1 is highly phosphorylated in many cancer tissues including breast cancer tissues. To understand the pathological functions of phosphorylated HSPB1 in cancer cells, we generated a phosphorylation-resistant mutant of HSPB1 (HSPB1-S3A) in which the phosphorylation sites at S15, S78, and S82 were substituted with alanine residues. HA-tagged wild-type HSPB1 and HSPB1-S3A were overexpressed in HEK293T cells and cell lysates were immunoblotted with antibodies that specifically recognize phosphorylated HSPB1 at each mutated site. We confirmed the specificity of these antibodies and examined the phosphorylation levels of wild-type HSPB1 (HSPB1-WT) and mutant HSPB1-S3A in MCF-7 cells (Fig. S3*A*). We observed reduced phosphorylated HSPB1 in the crude cell lysate expressing HSPB1-S3A compared to the crude cell lysate expressing HSPB1-WT in both wild-type and HSPB1-KD MCF-7 cells (Figs. 3*A* and S3*B*). The HSPB1-S3A mutant localized to both the cytoplasm and membrane, similar to the intracellular localization of HSPB1-WT in ER+ breast cancer cells (Fig. 3*B*). Surprisingly, the HSPB1-S3A mutant showed impaired growth-promoting ability compared with wild-type HSPB1 (Figs. 3*C* and S3*C*). Furthermore, HSPB1 expression in HSPB1-KD T47D cells was compensated by expressing shRNA-resistant wild-type HSPB1 or HSPB1-S3A mutant and the cell proliferation ability was assessed. The growth defect in HSPB1-KD cells was partially rescued by expressing shRNA-resistant wild-type HSPB1 (Fig. S3, *D* and *E*). However, the HSPB1 mutant could not rescue the growth defect by HSPB1-KD to the same extent as wild-type HSPB1 (Fig. S3, *D* and *E*), suggesting that HSPB1 positively regulates cell proliferation *via* phosphorylation-dependent and phosphorylation-independent ways in T47D cells.

To examine the chaperone activity of the HSPB1-S3A mutant, we conducted another interactome analysis. HA-tagged wild-type HSPB1 and mutant HSPB1-S3A were expressed in MCF-7 cells, and cell lysates were immunoprecipitated with an anti-HA antibody. The precipitates were then analyzed using mass spectrometry. While 4062 binding partners were detected in the HSPB1-WT sample, 846 interactants were noted in the HSPB1-S3A sample. SLC7A5 was detected in the HSPB1-WT dataset but remained undetected in the HSPB1-S3A dataset (Fig. 3*D* and Table S3). No significant difference could be observed in the detected HSPB1 protein levels in the proteomic analysis between HA-tagged HSPB1-WT and HSPB1-S3A samples, suggesting that phosphorylation-resistant HSPB1 decreased the chaperoning activity (Fig. S3*F*). In addition, the HSPB1-SLC7A5 interaction was impaired in the HSPB1-S3A mutant (Figs. 3*E* and S3*G*)

To understand which phosphorylation site is responsible for the SLC7A5 complex formation, we prepared HSPB1-knockout (KO) HEK293T cells using the CRISPR/Cas9 system (Fig. 3*F*). HSPB1-KO did not change the cell proliferation ability in HEK293T cells (Fig. 3*F*). Using the HSPB1-KO cells, the complex formation among HSPB1, SLC7A5, and LLGL2 was examined. We expressed the wild-type HSPB1, HSPB1-S3A, and HSPB1-S3D, which three serine residues are substituted with aspartate, and the interaction was investigated. HSPB1-S3A reduced the LLGL2-SLC7A5 and LLGL2-HSPB1 interaction down to the background levels (Fig. 3*G*). In addition, HSPB1-S3D retains LLGL2-SLC7A5 interaction at similar levels with wild-type HSPB1 expression, suggesting that phosphorylated HSPB1 requires for the LLGL2-SLC7A5 complex formation (Fig. 3*G*). Furthermore, we prepared the single and double phosphor-resistant mutant of HSPB1 and expressed in HSPB1-KO cells. The cell lysates were immunoprecipitated with anti-HA antibody. Both single and double phosphorylation resistant mutants could not interact with SLC7A5 and LLGL2, suggesting that phosphorylation at three serine residues, that is at S15, S78, and S82, is required for the complex formation with SLC7A5 and LLGL2 (Figs. 3*H* and S3*H*).

### Nutrient stress and amino acid deficiency induce HSPB1 phosphorylation in ER+ breast cancer cells

**Figure 4:**
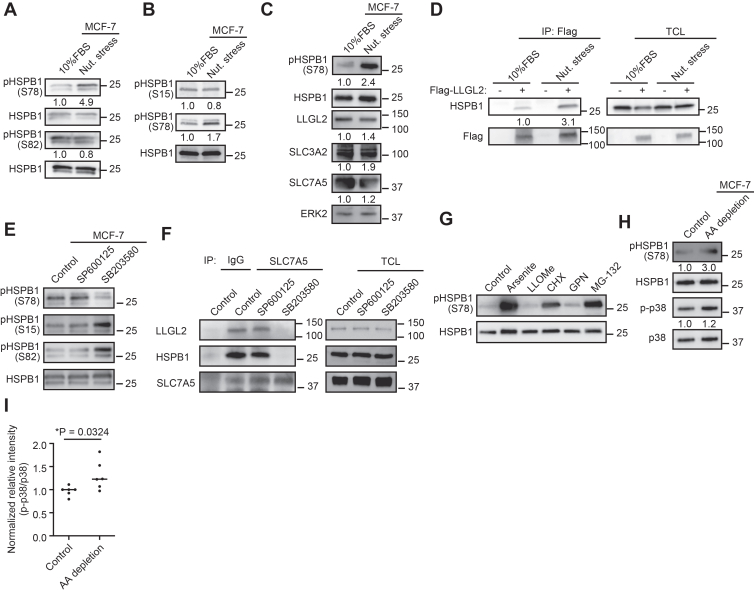
**HSPB1 is phosphorylated at S78 residue by p38 under nutrient-stressed condition.***A, B*, the comparison of phosphorylation levels of HSPB1 at Ser 78 (*A*, *B*), Ser 82 (*A*), and at Ser 15 (*B*) under normal culture medium (10% FBS supplemented medium) and nutrient stress culture medium (See materials and methods). *C*, protein levels of LLGL2, SLC3A2, and SLC7A5 under normal culture medium and nutrient stress medium. *D*, immunoprecipitation of endogenous HSPB1 and Flag-tagged LLGL2 in MCF-7 cells cultured in different conditions. *E*, MCF-7 cells were treated with 1 μM JNK (SP600125) or p38 (SB203580) inhibitor for 1 day and the phosphorylation levels of HSPB1 at S78, S15, and S82 were examined by immunoblot. *F*, The cell lysates of 1 μM JNK or p38 inhibitor-treated MCF7 cells were immunoprecipitated with anti-SLC7A5 antibody, and the precipitates were immunoblotted with the indicated antibodies. *G*, Arsenite, LLOMe, CHX, GPN, and MG-132 were treated to induce cellular stress in MCF-7 cells. Each compound was treated with 100 μM at the final concentration. The phosphorylation levels of HSPB1 at S78 were investigated by immunoblotting. *H*, amino acid-depletion activates p38 in MCF-7 cells. *I*, The signal intensity of p-p38 were quantitated. Data *I*, is shown as mean with each plots, *n* = 5 (*I*). Statistical analysis was conducted by two-tailed *t* test (*I*).

Based on our previous finding that SLC7A5’s association with LLGL2 is enhanced under nutrient stress conditions (30), we compared the phosphorylation status of HSPB1 in both 10% FBS-supplemented and serum-free culture media. Serum-free culture medium was referred to as the nutrient stress medium in our previous report (13). After 2 days of culture in the media, we assessed the phosphorylation levels of HSPB1 at S15, S78, and S82. Interestingly, the phosphorylation levels of HSPB1 at S78 were consistently upregulated under nutrient stress conditions among the ER+ breast cancer cell lines examined (Figs. 4, *A*, *B* and S4, *A*, *B*). Nutrient stress culture condition did not downregulate the protein levels of LLGL2, SLC3A2, and SLC7A5 in MCF-7 cells (Fig. 4*C*). Furthermore, nutrient stress augmented the LLGL2-HSPB1 interaction compared to the culture medium supplemented with 10% FBS, consistent with the phenotype of the LLGL2-SLC7A5 interaction (Figs. 4*D* and S4*C*).

Although several kinases that phosphorylate HSPB1 have been reported, neither the kinase that phosphorylates HSPB1 in response to nutrient stress nor the kinase that phosphorylates HSPB1 in ER+ breast cancer has been elucidated. Recent report showed that p38 stress MAPK phosphorylates HSPB1 at S15, S78, and S82 residues (36). Thus, we investigated the involvement of stress MAPK pathway. We treated SP600125 as a JNK inhibitor and SB203580 as a p38 inhibitor, and the phosphorylation levels of HSPB1 were examined. We found that p38 inhibition strongly suppressed the phosphorylation of HSPB1 at Ser 78, but not at Ser 15 or Ser 82, in MCF-7 and T47D cells (Figs. 4*E* and S4*D*). Furthermore, p38 inhibition strongly suppressed the SLC7A5-LLGL2 and SLC7A5-HSPB1 interactions, supporting phosphor-resistant HSPB1 mutant experiments (Fig. 4*F*).

Next, we attempted to understand the cellular stress that increases HSPB1 phosphorylation at S78 in ER+ breast cancer cells. MCF-7 and T47D cells were treated with sodium arsenite (oxidative stress), L-Leucyl-L-Leucine methyl ester (LLOMe; lysosomal stress), cycloheximide (CHX; translational stress), Gly-Phe-β-naphthylamide (GPN; lysosomal stress), and MG-132 (proteasomal stress). Sodium arsenite and MG-132 treatment increased phosphorylation levels of HSPB1 at Ser 78 (Figs. 4*G* and S4*E*). Given that SLC7A5 facilitates amino acid uptake and MG-132 consistently increased phosphorylation of HSPB1 at S78 residue, we examined the effect of amino acid depletion on HSPB1 phosphorylation at S78. Interestingly, amino acid depletion increased phosphorylation of HSPB1 at S78 in MCF-7, T47D, ZR75-1, and Hs578T cells (Figs. 4*H* and S4, *F*–*H*). Furthermore, amino acid depletion activated p38, suggesting that p38-mediated HSPB1 phosphorylation senses amino acid-depleted condition to promote membranous SLC7A5 in breast cancer cells (Figs. 4, *H*, *I* and S4, *I*, *J*) although further investigation is required to understand the molecular mechanisms by which amino acid depletion activates p38.

### HSPB1-SLC7A5 confers paclitaxel resistance in ER+ breast cancer cells

**Figure 5:**
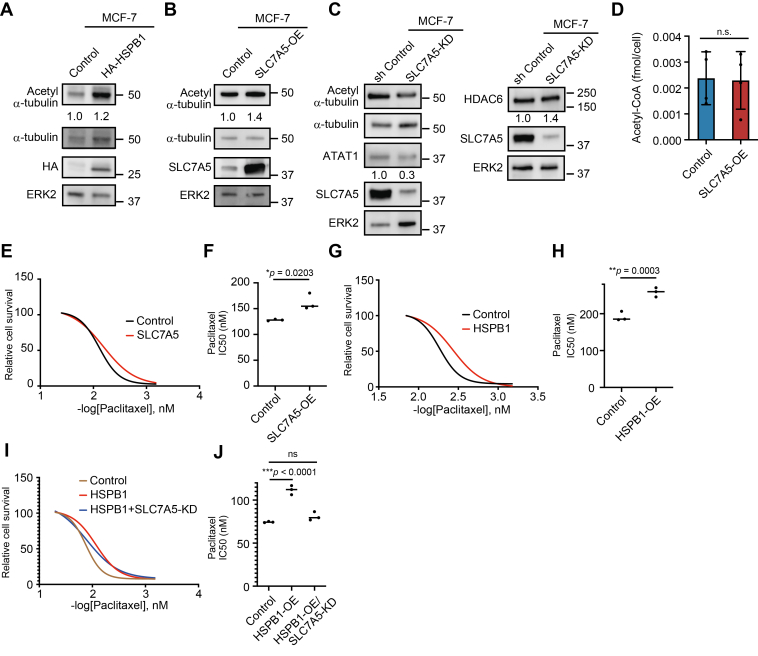
**HSPB1-SLC7A5 induces paclitaxel resistance in ER+ breast cancer cells.***A*, acetylated α-tubulin levels in HSPB1-OE MCF-7 cells. *B*, acetylated α-tubulin levels in SLC7A5-OE MCF-7 cells. *C*, SLC7A5-KD downregulates ATAT1 and upregulates HDAC6 in MCF-7 cells. *D*, acetyl-CoA concentration was measured by metabolome analysis in SLC7A5-OE MCF-7 cells. *E*, The dose response curve of paclitaxel-treated SLC7A5-OE and control MCF-7 cells. *F*, IC50 value of paclitaxel-treated SLC7A5-OE MCF-7 cells. *G*, The dose response curve of paclitaxel-treated HSPB1-OE and control MCF-7 cells. *H*, IC50 value of paclitaxel-treated HSPB1-OE MCF-7 cells. *H*, IC50 value of paclitaxel-treated HSPB1-OE cells. *I*, IC50 value of paclitaxel-treated HSPB1-OE and HSPB1-OE/SLC7A5-KD MCF-7 cells. Data (*D*) is shown as mean ± s.d., Data (*F*, *H*, and *J*) are shown as mean with each plot; (*D*) *n* = 4, F; *n* = 3, H; *n* = 3, J; *n* = 3. Statistical analysis was conducted by two-tailed *t* test (*D*, *F*, *H*) or one-way ANOVA followed by Tukey’s post-test (*J*).

We observed that HSPB1-OE and SLC7A5-OE cells upregulated acetylated α-tubulin levels (Figs. 5, *A* and *B*). Tubulin is acetylated by alpha tubulin acetyltransferase 1(ATAT1) and deacetylated by histone deacetylase 6 (HDAC6) (37). We examined ATAT1 and HDAC6 protein levels in SLC7A5-KD cells. SLC7A5-KD downregulated ATAT1 and upregulated HDAC6 in MCF-7 cells (Fig. 5*C*). In addition, SLC7A5-OE did not change the intracellular acetyl-CoA, which becomes a substrate of protein acetylation (Fig. 5*D*). However, further investigation will be needed to understand how HSPB1-SLC7A5 regulates tubulin acetylation in breast cancer cells. High levels of acetylated tubulin have been observed in cancer (38), and acetylation contributes to tubulin stability (39). Furthermore, acetylated tubulin enhances tolerance to paclitaxel-induced cell death in lung cancer (40). Therefore, we hypothesized that HSPB1-SLC7A5 affects paclitaxel tolerance, which stabilizes microtubules by inhibiting tubulin depolymerization in ER+ breast cancer cells. Indeed, SLC7A5 or HSPB1 overexpression induced paclitaxel resistance in MCF-7 cells (Fig. 5, *E*–*H*). Furthermore, HSPB1 overexpression conferred paclitaxel resistance, whereas SLC7A5-KD in HSPB1-OE cells lost their tolerance to paclitaxel (Fig. 5, *I* and *J*), indicating that HSPB1-SLC7A5 is essential for acquiring paclitaxel resistance in ER+ breast cancer cells.

### HSPB1-SLC7A5 induces tolerance to both tamoxifen and paclitaxel in ER+ breast cancer cells

**Figure 6:**
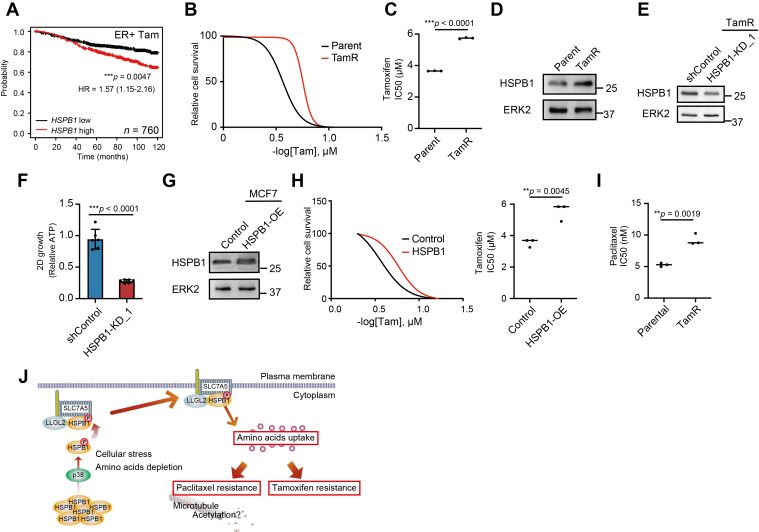
**HSPB1-SLC7A5 induces tolerance to both tamoxifen and paclitaxel in ER+ breast cancer cells.***A*, Kaplan-Meier plot of only tamoxifen-treated ER+ breast cancer patients. *B*, The dose response curve of tamoxifen-treated tamoxifen-resistant cells (TamR) and the parental MCF-7 cells. *C*, IC50 values of tamoxifen-treated parental and tamoxifen-resistant cells. *D*, HSPB1 expression in tamoxifen-resistant cells. *E*, Knockdown of HSPB1 in tamoxifen-resistant cell (TamR). *F*, 2D growth of HSPB1-KD TamR cells. *G*, HSPB1 overexpression in MCF7 cells. *H*, IC50 values of tamoxifen-treated HSPB1-OE cells. *I*, IC50 values of paclitaxel-treated TamR cells. *J*, Model of drug resistance acquisition by HSPB1-SLC7A5 pathway. Data (*C*, *F*, *H*, and *I*) are shown as mean ± s.d.; Data *B* and *H* are shown as mean. *C, H, I*; *n* = 3, *F*, *n* = 6. Statistical analysis was conducted by two-tailed Student’s *t* test (*C*, *F*, *H*, *I*).

Given that SLC7A5 upregulation results in tamoxifen resistance and HSPB1 is essential for the SLC7A5 function in ER+ breast cancer cells (13), we investigated whether HSPB1 is associated with tamoxifen resistance in ER+ breast cancer cells. High *HSPB1* mRNA levels were found to be correlated with poor clinical prognosis in ER+ patients treated with tamoxifen (Fig. 6*A*). We have established tamoxifen-resistant (TamR) MCF-7 cells, and the IC50 is shown in Figure 5, *B* and *C*. We observed HSPB1 upregulation in HSPB1 protein levels (Fig. 6*D*). Next, we knocked down HSPB1 in tamoxifen-resistant (TamR) cells using RNA interference (Fig. 5*E* and S5*A*). HSPB1-KD inhibited the proliferation of tamoxifen-resistant cells (Figs. 6*F* and S5*B*). Intriguingly, HSPB1-OE induced tamoxifen resistance in the ER+ breast cancer cell lines, MCF-7 and T47D (Figs. 6, *G*, *H* and S5*C*), indicating that HSPB1 plays a pivotal role in tamoxifen-resistant cell proliferation and acquisition of tamoxifen resistance in ER+ breast cancer cells. Furthermore, paclitaxel-resistance is acquired in tamoxifen-resistant cells (Fig. 6*I*). These findings suggest the novel pathological role of HSPB1-SLC7A5 in drug resistance. Therefore, the HSPB1-SLC7A5 complex is a critical axis for therapeutic evasion in breast cancer (Fig. 6*J*).

## Discussion

Cancer cells modify nutrient uptake in response to environmental nutrient conditions, particularly under nutrient-stress conditions. SLC7A5 is a fundamental amino acid transporter that is used for amino acid incorporation into cells (12), but how SLC7A5 activity is controlled in response to nutrient stress has not been understood. SLC7A5 functions are well known to be regulated by gene expression and intracellular localization. Most cancer cells overexpress SLC7A5. Thus, this study uncovered the molecular mechanisms of how SLC7A5 intracellular localization in response to nutrient stress. We identified a chaperone molecule, HSPB1, which is a key regulator of SLC7A5 regulation under nutrient stress and first revealed a novel metabolic HSPB1 function in ER+ breast cancer cells. Importantly, HSPB1 promotes cell proliferation and serves as an essential component of the SLC7A5-LLGL2 complex, and its loss disrupts SLC7A5-LLGL2 interaction, resulting in reduced leucine uptake in ER+ breast cancer cells. HSPB1 overexpression did not promote cell proliferation in Hs578T and BT549 cells, but HSPB1 knockdown suppressed cell proliferation in those cells. Further investigation will be required to understand the phenotypes of HSPB1-OE in those cells.

Furthermore, we demonstrated how HSPB1 contributes to the adoption of environmental nutrient conditions. We showed that HSPB1 phosphorylation is essential for the SLC7A5 association in ER+ breast cancer. Our HSPB1 mutant analysis revealed that phosphorylation at S15, S78, and S82 residues is needed for the complex formation with SLC7A5. The phosphorylation levels of S15, S78, and S82 are different in MCF-7 in our culture conditions. We observed the upregulation of the S78 residue under nutrient stress conditions. In addition, we found that p38 is an HSPB1 kinase at S78 in ER+ breast cancer cells, responding to amino acid depletion. Considering that phosphorylation levels of HSPB1 at S15 and S82 residues were not changed by nutrient stress, we believe that Serine 78 residue in HSPB1 functions as a nutrient sensor to augment amino acid uptake, although further investigation is warranted to prove that HSPB1 functions as a nutrient sensor.

Downregulation of HSPB1, SLC7A5, and LLGL2 also reduced the acetylated α-tubulin in ER+ breast cancer cells. SLC7A5 is critical for maintaining normal brain branched-chain amino acid (BCAA) levels, and deletion of SLC7A5 from the endothelial cells of the blood–brain barrier (BBB) leads to severe neurological abnormalities in mice (41, 42). Given these previous reports and our results, depletion of SLC7A5-mediated BCAA uptake can lead to the decrease of acetylated α-tubulin, resulting in neurological abnormalities. However, further analysis is needed to elucidate the relationship between SLC7A5-mediated BCAA uptake and acetylated α-tubulin levels.

Overexpression of HSPB1 and SLC7A5 elevates acetylated tubulin level. Tubulin acetylation has been correlated with paclitaxel tolerance in lung cancer cells, although the pathological significance of microtubule stability and the direct molecular mechanism that connects tubulin acetylation and paclitaxel resistance is not entirely understood (40). We examined the paclitaxel sensitivity in SLC7A5-OE cells. Intriguingly, SLC7A5 overexpression induced paclitaxel resistance and SLC7A5-KD in HSPB1-OE cells sensitized to paclitaxel, thereby revealing a novel pathological role of HSPB1-SLC7A5 in ER+ breast cancer cells, and the HSPB1-mediated paclitaxel resistance relies on SLC7A5 function.

Given that aberrant HSPB1 expression is associated with therapeutic resistance in tumors, we hypothesized that the HSPB1-SLC7A5 axis plays an essential role in drug resistance in cancer cells. In our previous study, SLC7A5 upregulation was shown to induce tamoxifen resistance (30). Hence, we assessed the effect of HSPB1 on the endocrine therapy drug tamoxifen. High levels of HSPB1 mRNA are associated with poor clinical prognosis in tamoxifen-treated patients with breast cancer, and HSPB1 is essential for tamoxifen-resistant cell proliferation. Furthermore, HSPB1 overexpression induces tamoxifen resistance in ER+ breast cancer cells, underscoring the importance of HSPB1-SLC7A5 in both tamoxifen resistance and paclitaxel resistance.

Our observations identified a hitherto unknown role of the heat shock protein HSPB1 in regulating SLC7A5 function in cancer cells. This newfound function of HSPB1 represents a novel molecular mechanism linking its pathological role to abnormal amino acid metabolism. By analyzing the role of HSPB1 in ER+ breast cancer cells, we revealed the crucial pathological role of SLC7A5 in acquiring paclitaxel resistance. Considering that the HSPB1-SLC7A5 complex also leads to tamoxifen resistance, we unveiled the HSPB1-SLC7A5 axis as a pivotal factor for drug resistance in ER+ breast cancer cells.

## Experimental procedures

### Cell lines

MCF-7, T47-D, ZR-75-1, BT-474, AU565, HCC1569, MDA-MB 231, Hs578T, BT-549, and HEK293T cell lines were obtained from ATCC. MCF-7 cells were cultured in EMEM (Nacalai) supplemented with 10% FBS, 10 μg/ml insulin, 1 mM sodium pyruvate, and penicillin-streptomycin (Nacalai). T47D cells were cultured in RPMI1640 (Nacalai) supplemented with 10% FBS, 5 μg/ml insulin, and penicillin-streptomycin (Nacalai). BT474, AU565, HCC1569, and BT-549 cells were cultured in RPMI1640 (Nacalai) supplemented with 10% FBS and penicillin-streptomycin (Nacalai). MDA-MB 231, Hs578T, and HEK293T cells were cultured in DMEM (Nacalai) supplemented with 10% FBS and penicillin-streptomycin (Nacalai). All cell lines were monitored by MycoAlert PLUS (Lonza) and maintained in mycoplasma-free conditions.

### Antibodies and reagents

Anti-LLGL2 (A-4), anti-HSP27 (F-4), anti-α-tubulin (B-7), anti-a-tubulin (DM1A), anti-acetylated α-tubulin (6-11B-1), and anti-ERK2 (D-2) antibodies were purchased from Santacruz biotechnology. Anti-HA (C29F4); anti-pHSP27 S15 (#2404), S78 (#2405), and S82 (#2401); anti-SLC7A5 (E9O4D) and anti-E-cadherin (24E10) antibodies were purchased form Cell Signaling Technology. Anti-Flag (M2) and anti-HA (561-5) antibodies were purchased from MBL. Anti-HA (3F10) was purchased from Sigma. Anti-HDAC6 (12834-1-AP) and anti-ATAT1 (28828-1-AP) were purchased from Proteintech. 4-OH tamoxifen was purchased from Sigma (H7904-5MG). Paclitaxel was purchased from Nacalai (19879-66). Sodium arsenite was purchased from Sigma. LLOMe, CHX, GPN, and MG-132 were purchased from Cayman chemical.

### Immunoblot

Western blot was conducted as previously described (43). Briefly, cells were lysed with lysis buffer (50 mM Tris-HCl, pH 7.5, 150 mM NaCl, 5 mM EDTA, 1% Triton X-100, protease inhibitors, and phosphoSTOP tablets (Roche)). The supernatant was subject to SDS-PAGE, and proteins were transferred onto the PVDF membrane (Millipore #IPVH00010). After blocking with 1% BSA/TBS-0.1% Tween 20, the primary antibody (1:1000 dilution) was reacted overnight at 4 °C. HRP-conjugated secondary antibodies (1:10,000 dilution; MBL) and HRP substrate (Pierce) were used for detection. Images were captured using LAS-4000 mini (Fujifilm) or ChemiDoc Touch (Bio-Rad). The quantification of signal intensity was conducted using ImageJ64 software or Image Lab (Bio-Rad).

### Construction of viral vectors

The cDNA of HSPB1 was obtained from Dharmacon. HSPB1 cDNA was subcloned into pLJM1 vector (Addgene #19319) at AgeI and EcoRI restriction enzyme sites using the NEBuilder HiFi DNA Assembly system (NEB #E2621L). We prepared plenti-CAG-IRES-Puro usingplenti-CAG-IRES-GFP (Addgene #122953). HSPB1 cDNA was subcloned into plenti-CAG-IRES-Puro at BamHI restriction enzyme site using the NEBuilder HiFi DNA Assembly system (NEB #E2621L). Briefly, PCR primers were designed using NEBuilder Assembly Tool (https://nebuilder.neb.com), and PCR was performed using KOD-plus (TOYOBO) following the manufacturer’s instructions. Digested pLJM1 or plenti-CAG-empty-IRES-EGFP vector and purified PCR fragment were assembled using NEBuilder HiFi DNA Assembly (NEB #E2621L) following the manufacture’s instruction.

The shRNA for targeting HSPB1 (#1: TRCN0000008753 5′-CCGATGAGACTGCCGCCAAGTCTCGAGACTTGGCGGCAGTCTCATCGG-3′ and #2: TRCN0000356587 5′- CAGTCCAACGAGATCACCATCCTCGAGGATGGTGATCTCGTTGGACTG-3′), for targeting SLC7A5 (TRCN0000043009 5′-CTAGATCCCAACTTCTCATTT-3′), and for non-targeting control shRNA (SHC002 5′-CAACAAGATGAAGAGCACCAA-3′) are subcloned into pLKO.1-blast (Addgene #26655) at AgeI and EcoRI restriction enzyme sites. For CRISPR/Cas9 sysmtem, we targeted two different HSPB1 sequence (5′-GAGTGGTCGCAGTGGTTAGG-3′ and 5′-GGATGTCAACCACTTCGCCC-3′) and each gRNAs were cloned into lentiCRISPRv2 (addgene#52961) following the cloning protocol.

The shRNA-resistant cDNA for shHSPB1 #1 (TRCN0000008753) and shHSPB1 #2 (TRCN0000356587) were generated by mutating the target sequence. The sequences are 5′-cTgaCgaAacGgcAgcAaaAt-3′ for shHSPB1 #1 and 5′- caAAGTaaTgaAatAacAatA-3′ for shHSPB1 #2. In addition, the CRISPR-Cas9 resistant HSPB1 cDNA was generated by mutating the sequences of gRNA targeting site (from 5′-GAGTGGTCGCAGTGGTTAGG-3′ to 5′- gaAtggtcAcaAtggCTGgg-3′, and from 5′-GGATGTCAACCACTTCGCCC-3′ to 5′-AgaCgtTaaTcaTttTgcAc-3′).

### Generation of viral vectors

The procedures for lentivirus and retrovirus production were described previously (14). Briefly, each lentivirus was produced by transfection with pCMV dR8.91 and pCMV-VSV-G (Addgene #8454) by calcium phosphate transfection in HEK293T cells. After 2 days from transfection, the supernatant was collected and filtered with a 0.45 μm diameter PES syringe filter. The supernatant was aliquoted into tubes and stored at −80 °C until use.

### 2D cell proliferation assay

One million cells were spread on a 6 cm culture dish. The next day, the culture medium was changed to DMEM/F12 (Nacalai #08460-95) supplemented with 20 ng/ml EGF (Peprotech AF-100-15) and 1x B27 supplement minus vitamin A (Thermo Fisher Scientific #12587010). Simultaneously, we collected cells by trypsinization and counted the cell numbers using Countess 3 Automated Cell Counter (Invitrogen) with 0.4% trypan blue staining. We set this time point as day 0. At the indicated time points, the cell number was counted. The results were confirmed with more than two independent experiments with three biological replicates.

To evaluate the dead cell number, cells were collected by trypsinization at indicated timepoint and the collected cells were stained with 0.4% trypan blue staining solution. Living and dead cell numbers were counted using Countess 3 Automated Cell Counter (Invitrogen).

### Cell viability assay

Cells were plated on 96-well plates at 10,000 cells per well. The next day, the medium was changed to DMEM/F12 supplemented with 1× B27 minus vitamin A and 20 ng/ml EGF. After 4 days of incubation, the cell viability assay was performed using CellTiter-Glo 3D (Promega) following the instructions. The luminescence intensity was measured by a plate reader (TECAN infinite M200). The results were confirmed with three independent experiments with six biological replicates.

### Nano-BiT assay

The membrane localization of SLC7A5, SLC3A2, and LLGL2 was assessed by Nano-glo cell viability assay (Promega). The PH domain of PLCdelta was fused with LgBiT was cloned into pLJM1 vector. In addition, SmBiT-SLC7A5, SmBiT-SLC3A2, or LLGL2-SmBiT was cloned into pInducer13 (Addgene #46936) at AgeI and NotI sites. The tet-inducible SmBiT-SLC7A5, SmBiT-SLC3A2, or LLGL2-SmBiT was co-expressed with PH-PLCD-LgBiT in MCF-7 cells.

Thousands of cells were spread into a 384-well white plate in the presence or absence of 2 μg/ml doxycycline in duplicate. After 16 h of culture, the membrane localization of SLC7A5, SLC3A2, and LLGL2 were assessed by using the nano-glo cell viability assay (Promega). The other plate was applied for Celltiter Glo assay (Promega) following the instructions. The signal intensity of Nano-glo assay was normalized with the signal intensity of Celltiter Glo assay.

### Immunofluorescence staining

Cells were plated on cover slips (Matsunami glass #C018001) and cultured in 12-well plate. Cells were fixed with 2% formalin/PBS for 15 min at room temperature. The slides were washed with PBS twice and permeabilized with 0.02% saponin/PBS for 20 min. The slides were washed with PBS twice, and cells were incubated in a blocking solution (1% FBS/1% BSA in PBS) for 1 h at room temperature. Cells were treated with primary antibody solution for overnight at 4 °C. After three times wash with PBS, cells were treated with Alexa Fluor-conjugated secondary antibody solution for 1 h at room temperature. Cells were counter-stained with DAPI, and coverslips were enclosed with anti-fade regent (Vector laboratories). The images were captured using a confocal microscope (Carl Zeiss LSM900).

### Immunoprecipitation

Cells were collected and lysed with lysis buffer (50 mM Tris pH7.4, 100 mM NaCl, 5 mM EDTA pH8.0, 1% Triton-X, 2 mM sodium orthovanadate, 1 mM NaF, 1 mM beta-glycerophosphate, cOmplete (Roche) and phosSTOP (Roche). Cell lysates were pre-cleared with 20 μl of Protein G Sepharose beads (Cytiva #17061801) for 5 min and the cleared lysates were transferred to a new tube. Three μl of anti-HA antibody (Cell Signaling Technology #3724 or 3F10 Sigma) was added into cell lysates and incubated for 45 min at 4 °C with rotation. After washing 3 to 5 times with lysis buffer, precipitates were dissolved in 1x SDS sample buffer. The precipitates were analyzed with immunoblot.

### Survival analysis

Kaplan–Meier plots were analyzed using Kaplan-Meier Plotter (http://kmplot.com/analysis/).

### Biotin-labeling of surface proteins and pulldown assay

Cells were rinsed with ice-cold PBS, and surface proteins were labeled with 400 μM EZ-link-sulfo-NHS-SS-Biotin/PBS for 30 min at 4 °C. The reaction was quenched with 2 ml of 150 mM glycine. After twice rinse with ice-cold PBS, cells were lysed in lysis buffer (50 mM Tris (pH 7.4), 100 mM NaCl, 5 mM EDTA (pH 7.4), 1% Triton-X-100, 5 mM NaF, plus protease inhibitors and phosphoSTOP tablets (Roche)). 50 μg of protein lysate was incubated with 20 μl of Streptavidin Sepharose High Performance Beads (GE Healthcare) and rotated for 1.5 h at 4 °C. Streptavidin beads were washed three times with lysis buffer, and proteins were eluted with 1× sample buffer.

### Proteomic analysis

Proteins were extracted with SDS sample buffer and were separated by SDS–PAGE. Proteins in gel were reduced with 10 mM dithiothreitol followed by alkylation with 50 mM iodoacetamide. Tryptic-digested peptides were eluted from gel and were purified with SDB-XC StageTip (44). NanoLC-MS/MS was conducted using an OrbiTrap Exploris mass spectrometer (Thermo Fisher Scientific) with an Vanquish Neo UHPLC. Peptides were separated with a C18 packed emitter column (Nikkyo Technos, Tokyo, Japan). MS data were acquired in the data-independent analysis (DIA) mode and were analyzed subjected to a search against the Uniprot Human database with DIA-NN 1.8.1 (45). *p*-value cutoff and effect size cutoff were used to define enriched proteins as follows: *p*-value < 0.05 and fold change ≥ 2.

### Metabolome analysis by capillary electrophoresis mass spectrometry

#### Instrumentation

All capillary electrophoresis time-of-flight mass spectrometry (CE-TOFMS) experiments were performed using an Agilent CE capillary electrophoresis system (Agilent Technologies), an Agilent G6230B LC/MSD TOF system (Agilent Technologies), an Agilent1260 Infinity 2 series binary HPLC pump, and the G1603A Agilent CE-MS adapter- and G1607A Agilent CE-ESI-MS sprayer kit. For system control and data acquisition we used the MassHunter workstation software version B.08.00 for Agilent CE-TOFMS.

#### CE-MS method

Briefly, to analyze cationic compounds, a fused silica capillary (50 μm i.d. × 100 cm) was used with 1 M formic acid as the electrolyte (46) 1. Approximately 5 nl of sample solution were injected at 50 mbar for 5 s, and 30 kV of voltage was applied. The capillary temperature was maintained at 20 °C and the sample tray was cooled below 5 °C. Methanol/water (50% v/v) containing 0.01 μM hexakis(2,2-difluoroethoxy)phosphazene was delivered as the sheath liquid at 10 μl/min. ESI-TOFMS was performed in positive ion mode, and the capillary voltage was set to 4 kV. A flow rate of heated dry nitrogen gas (heater temperature 300 °C) was maintained at 10 psig. In TOFMS, the fragmentor, skimmer, and Oct RFV voltage were set at 75V, 50V, and 500V, respectively. Automatic recalibration of each acquired spectrum was achieved using the masses of the reference standards (^13^C isotopic ion of a protonated methanol dimer, *m/z* 66.0631) and (protonated ion of hexakis(2,2-difluoroethoxy)phosphazene, *m/z* 622.0290). To identify metabolites, relative migration times of all peaks were calculated by normalization to the reference compound 3-aminopyrrolidine. The metabolites were identified by comparing their *m/z* values and relative migration times to the metabolite standards. Quantification was performed by comparing peak areas to calibration curves generated using internal standardization techniques with methionine sulfone. The other conditions were identical to those described previously (47).

To analyze anionic metabolites, a commercially available COSMO(+) (chemically coated with cationic polymer) capillary (50 μm i.d. × 105 cm) (Nacalai Tesque, Kyoto, Japan) was used with a 50 mM ammonium acetate solution (pH 8.5) as the electrolyte (48). Sample solution (30 nl) was injected at 50 mbar for 30 s, and −30 kV of voltage was applied. Methanol/5 mM ammonium acetate (50% v/v) containing 0.01 μM hexakis(2,2-difluoroethoxy)phosphazene was delivered as the sheath liquid at 10 μl/min. A platinum needle was used for anionic metabolite analysis (48).

ESI-TOFMS was performed in negative ion mode, and the capillary voltage was set to 3.5 kV. For TOFMS, the fragmentor, skimmer, and Oct RFV voltage were set at 100V, 50V, and 500V, respectively. Automatic recalibration of each acquired spectrum was performed using reference masses of standards, *i.e.*, (^13^C isotopic ion of deprotonated acetatic acid dimer, *m/z* 120.0383) and ([hexakis(2,2-difluoroethoxy)phosphazene + deprotonated acetic acid, *m/z* 680.0355). For anion analysis, trimesate and CAS were used as the reference and the internal standards, respectively. The other conditions were identical to those described previously (48).

#### Data analysis

CE-TOFMS raw data were analyzed using our proprietary software MasterHands (ver, 2.17.0.10). Briefly, the data processing for each experiment included data conversion, binning data into 0.02 *m/z* slices, baseline elimination, peak picking, integration, and elimination of redundant features to yield all possible peak lists. Data matrices were generated by an alignment process based on corrected migration times, and metabolite names were assigned to the aligned peaks by matching *m/z* and the corrected migration times of our standards library. Relative peak areas were calculated based on the ratio of peak area divided by that of internal standards, and metabolite concentrations were calculated based on the relative peak area between the sample and standard mixture.

#### IC50 calculation

Cells were plated into a 96-well plate at a cell density of 1 × 10^4^ or 5 × 10^3^ cells per well. After 24 h, the culture medium was replaced with the assay medium (DMEM/F12 supplemented with B27 minus vitamin A and 20 ng/ml EGF) containing a series of tamoxifen dilutions at final concentration of 0 nM, 100 nM, 500 nM, 750 nM, 1 μM, 2.5 μM, 5 μM, 7.5 μM, 10 μM, and 50 μM. On day 4, the cell viability assay was performed using CellTiter-Glo 3D (Promega) following the instruction. The luminescence intensity was measured by a plate reader (TECAN infinite M200). The values of IC50 were calculated using Prism 10 software.

For the paclitaxel, cells were plated into a 96-well plate at the cell density of 5 × 10^3^ cells per well. After 24 h, the culture medium was replaced with the assay medium as described above, containing a series of paclitaxel dilutions at final concentrations of 0 nM, 500 pM, 1 nM, 2 nM, 4 nM, 10 nM, 20 nM, and 40 nM. On day 4, the cell viability assay was performed using CellTiter-Glo 3D (Promega) following the instructions. The luminescence intensity was measured by a plate reader (TECAN infinite M200). The values of IC50 were calculated using Prism 10 software. The results were confirmed with more than two independent experiments with three biological replicates.

### Statistical analysis

All statistical analyses were performed using Prism 9 or 10 software. To assess the statistical significance of a difference between two treatments, we used a two-tailed Student’s *t* test. To assess the statistical significance of differences between more than two treatments, we used one-way or two-way ANOVA followed by Tukey’s or Sidak’s multiple comparison test.

## Animal experiments

All mouse experiments had been approved by the Laboratory Animal Center, Keio University School of Medicine (Protocol #A2024-008) and were carried out following the ‘Guide for the Care and Use of Laboratory Animals’.

## Data availability

All data supporting the findings of this study are available within the paper and its supplementary Information. Raw proteomic data files are deposited in jPOST (http://jpostdb.org; jPOST ID: JPST002973/PXD050431).

## Supporting information

This article contains supporting information.

Figures S1–S6.

Tables S1–S3.

## Conflict of interest

The authors declare that they have no conflicts of interest with the contents of this article.
