# Supplementary material for: The cellular stress sensor HSPB1 regulates the membrane localization of amino acid transporter SLC7A5 in breast cancer
Source: J Biol Chem. 2026 May 27;302(7):113197. doi: 10.1016/j.jbc.2026.113197 (PMC13311823; doi:10.1016/j.jbc.2026.113197)
Supplement: Supplementary Figure S1 [file mmc1.pdf]

Supplementary Fig. 1

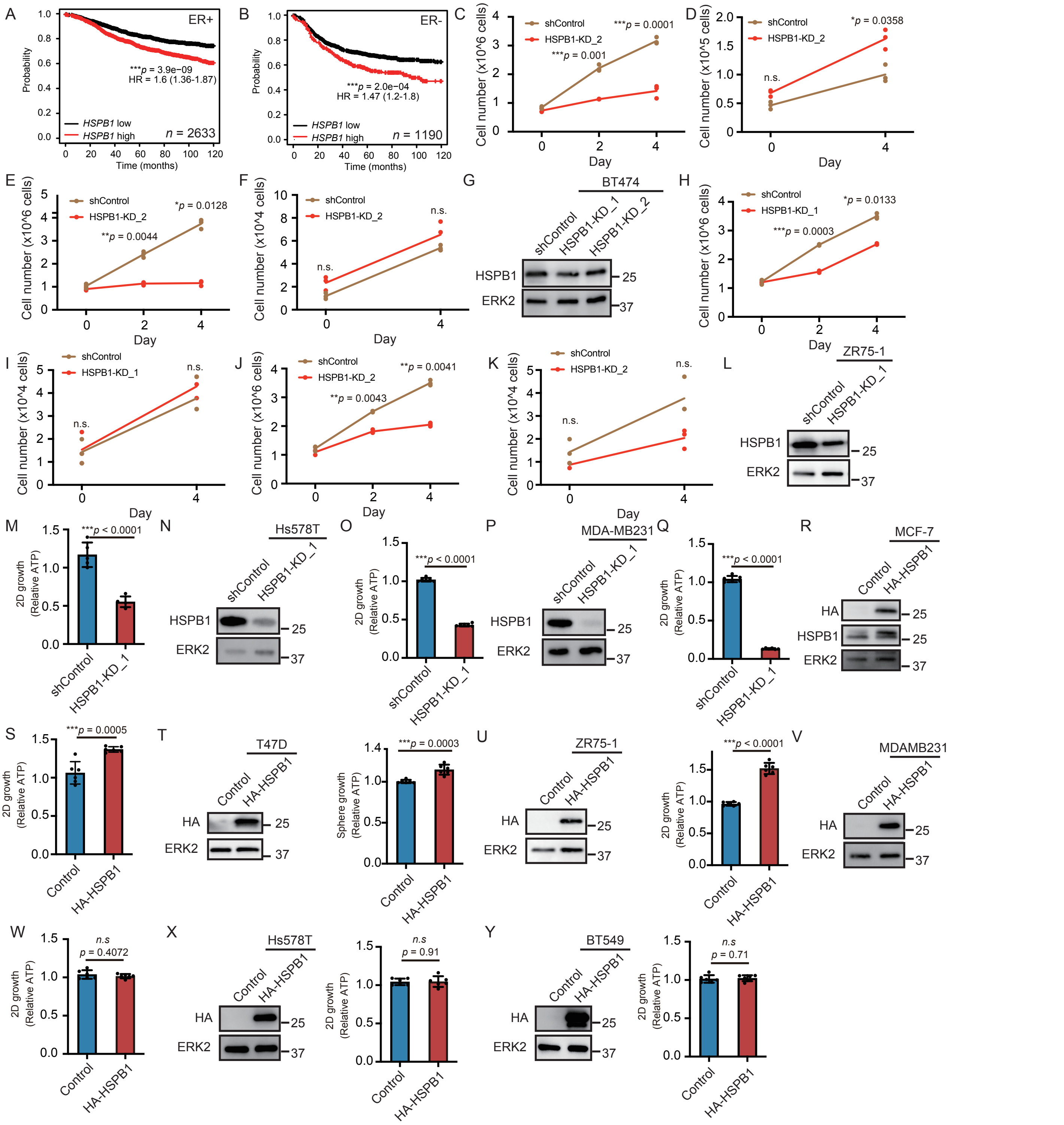

Supplementary Figure 1. HSPB1 regulates cell proliferation in ER+ breast cancer cells.

A, Kaplan-Meier plot of ER+ breast cancer patients. **B**, Kaplan-Meier plot of ER- breast cancer patients. **C**, The living cell number of HSPB1-KD MCF-7 cells. **D**, The dead cell number of HSPB1-KD cells. **E**, The living cell number of HSPB1-KD T47D cells. **F**, The dead cell number of HSPB1-KD T47D cells. **G**, HSPB1-KD in BT474 cells. **H**, The living cell number of HSPB1-KD BT474 cells. **I**, The dead cell number of HSPB1-KD BT474 cells. **J**, The living cell number of HSPB1-KD BT474 cells. **K**, The dead cell number of HSPB1-KD BT474 cells. **L**, HSPB1-KD in ZR75-1 cells. **M**, Relative cell number of HSPB1-KD ZR-75-1 cells by measuring intracellular ATP amount. **N**, HSPB1-KD in Hs578T cells. **O**, Relative cell number of HSPB1-KD Hs578T cells by measuring intracellular ATP amount. **P**, HSPB1-KD in MDA-MB 231 cells. **Q**, Relative cell number of HSPB1-KD MDA-MB 231 cells by measuring intracellular ATP amount. **R**, HA-HSPB1-expressing MCF-7 cells. **S**, Relative cell number of HA-HSPB1-expressing MCF-7 cells by measuring intracellular ATP amount. **T**, Immunoblot images of HA-HSPB1-expressing T47D cells (left). Relative cell number of HA-HSPB1-expressing T47D cells by measuring intracellular ATP amount (right). **U**, Immunoblot images of HA-HSPB1-expressing ZR75-1 cells (left). Relative cell number of HA-HSPB1-expressing ZR75-1 cells by measuring intracellular ATP amount (right). **V**, Immunoblot images of HA-HSPB1-expressing MDAMB231 cells. **W**, Relative cell number of HA-HSPB1-expressing MDAMB231 cells by measuring intracellular ATP amount. **X**, Immunoblot images of HA-HSPB1-expressing Hs578T cells (left). Relative cell number of HA-HSPB1-expressing Hs578T cells by measuring intracellular ATP amount (right). **Y**, Immunoblot images of HA-HSPB1-expressing BT549 cells (left). Relative cell number of HA-HSPB1-expressing BT549 cells by measuring intracellular ATP amount (right). Data **C, D, E, F, H, I, J, K, M, O, Q, S, T (right), U (right), W, X (right), and Y (right)** are shown as mean ± s.d; **C, D, E, F, H, I, J, K; n=3, M, O, Q, S, T (right), U (right), W, X (right), and Y (right)** n=6. Statistical analysis was conducted by two-tailed Student's t-test (**M, O, Q, S, T, U, W, X, Y**) and two-way ANOVA followed by Sidak's post-test (**C, D, E, F, H, I, J, K**).
