# Supplementary material for: The cellular stress sensor HSPB1 regulates the membrane localization of amino acid transporter SLC7A5 in breast cancer
Source: J Biol Chem. 2026 May 27;302(7):113197. doi: 10.1016/j.jbc.2026.113197 (PMC13311823; doi:10.1016/j.jbc.2026.113197)
Supplement: Supplementary Figure S2 [file mmc2.pdf]

Supplementary Fig. 2

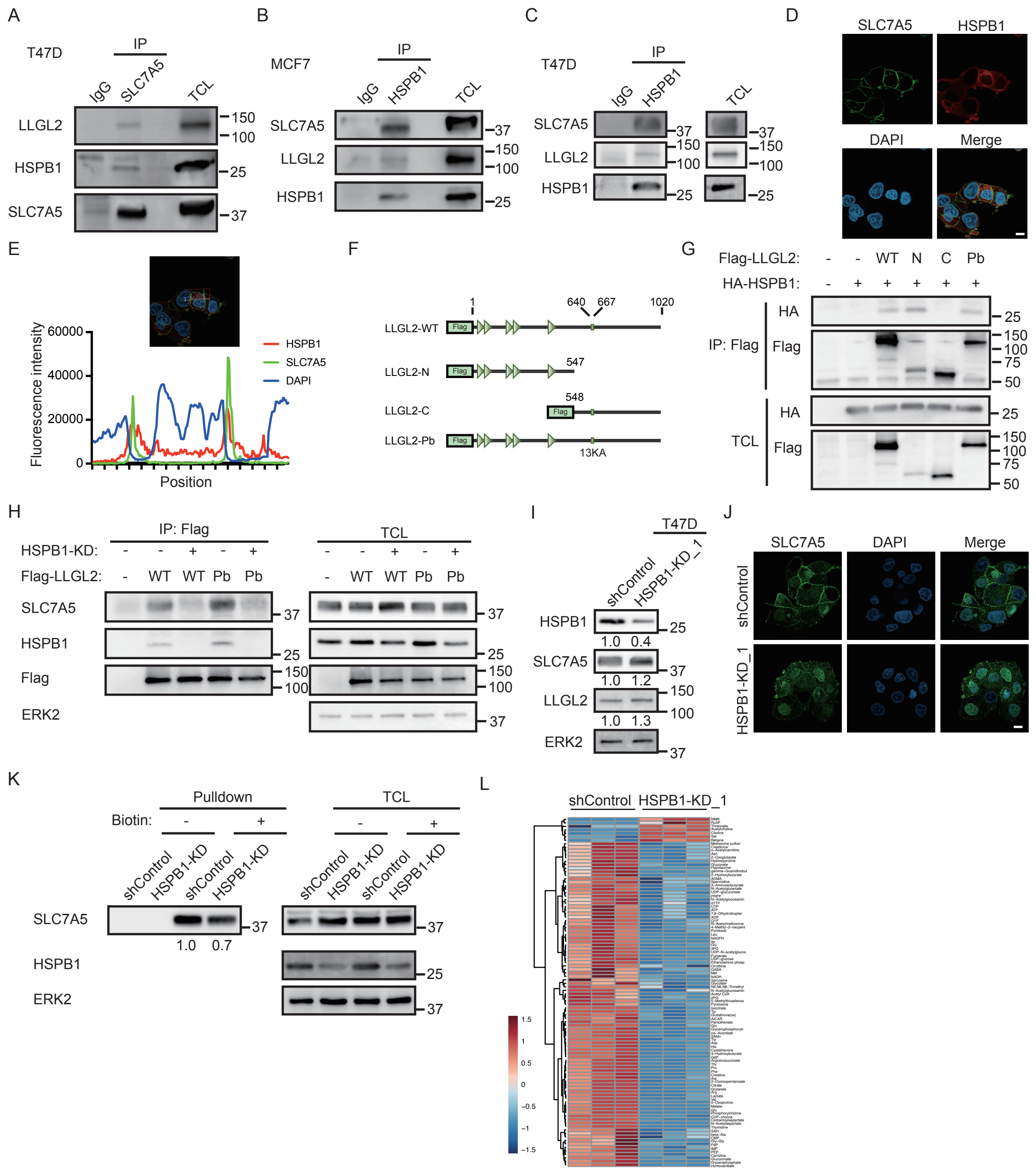

Supplementary Figure 2. HSPB1 regulates the plasma membrane localization of SLC7A5 in ER+ breast cancer cells.

A, HSPB1-SLC7A5 interaction at endogenous protein levels in T47D cells. The cell lysates were immunoprecipitated using anti-SLC7A5 antibody and the precipitates were immunoblotted with the indicated antibodies. **B**, HSPB1-SLC7A5 interaction at endogenous protein levels in MCF-7 cells. The cell lysates were immunoprecipitated using anti-HSPB1 antibody and the precipitants were immunoblotted with the indicated antibodies. **C**, HSPB1-SLC7A5 interaction at endogenous protein levels in T47D cells. The cell lysates were immunoprecipitated using anti-HSPB1 antibody and the precipitants were immunoblotted with the indicated antibodies. **D**, Immunostaining images of SLC7A5 (green), HSPB1 (red), and DAPI (blue). Scale bar indicates 10 μm. **E**, Quantification of signal intensity of the immunostaining image. The fluorescence intensity of the indicated area shown in an image was quantified. **F**, Schematic representation of LLGL2 constructs. **G**, HSPB1 interaction with LLGL2 mutants in wild-type HEK293T cells. The wild-type or mutant Flag-LLGL2 was expressed with or without HA-tagged wild-type HSPB1 in wild-type HEK293T cells. The cell lysates were immunoprecipitated with anti-Flag antibody and the precipitants were immunoblotted with the indicated antibodies. **H**, HSPB1 interaction with SLC7A5 in the presence of wild-type or Pb mutant LLGL2 in wild-type HEK293T cells. The wild-type or Pb mutant HSPB1 was expressed with or without shRNA targeting HSPB1 in wild-type HEK293T cells. The cell lysates were immunoprecipitated with anti-Flag and the precipitants were immunoblotted with the indicated antibodies. **I**, Immunoblot images of HSPB1-KD T47D cells. **J**, Immunostaining images of SLC7A5 (green) and DAPI (blue) in HSPB1-KD T47D cells. Scale bar indicates 10 μm. **K**, Surface protein levels of SLC7A5 in HSPB1-KD T47D cells. **L**, Metabolome analysis of HSPB1-KD T47D cells.
