# Supplementary material for: The cellular stress sensor HSPB1 regulates the membrane localization of amino acid transporter SLC7A5 in breast cancer
Source: J Biol Chem. 2026 May 27;302(7):113197. doi: 10.1016/j.jbc.2026.113197 (PMC13311823; doi:10.1016/j.jbc.2026.113197)
Supplement: Supplementary Figure S3 [file mmc3.pdf]

Supplementary Fig. 3

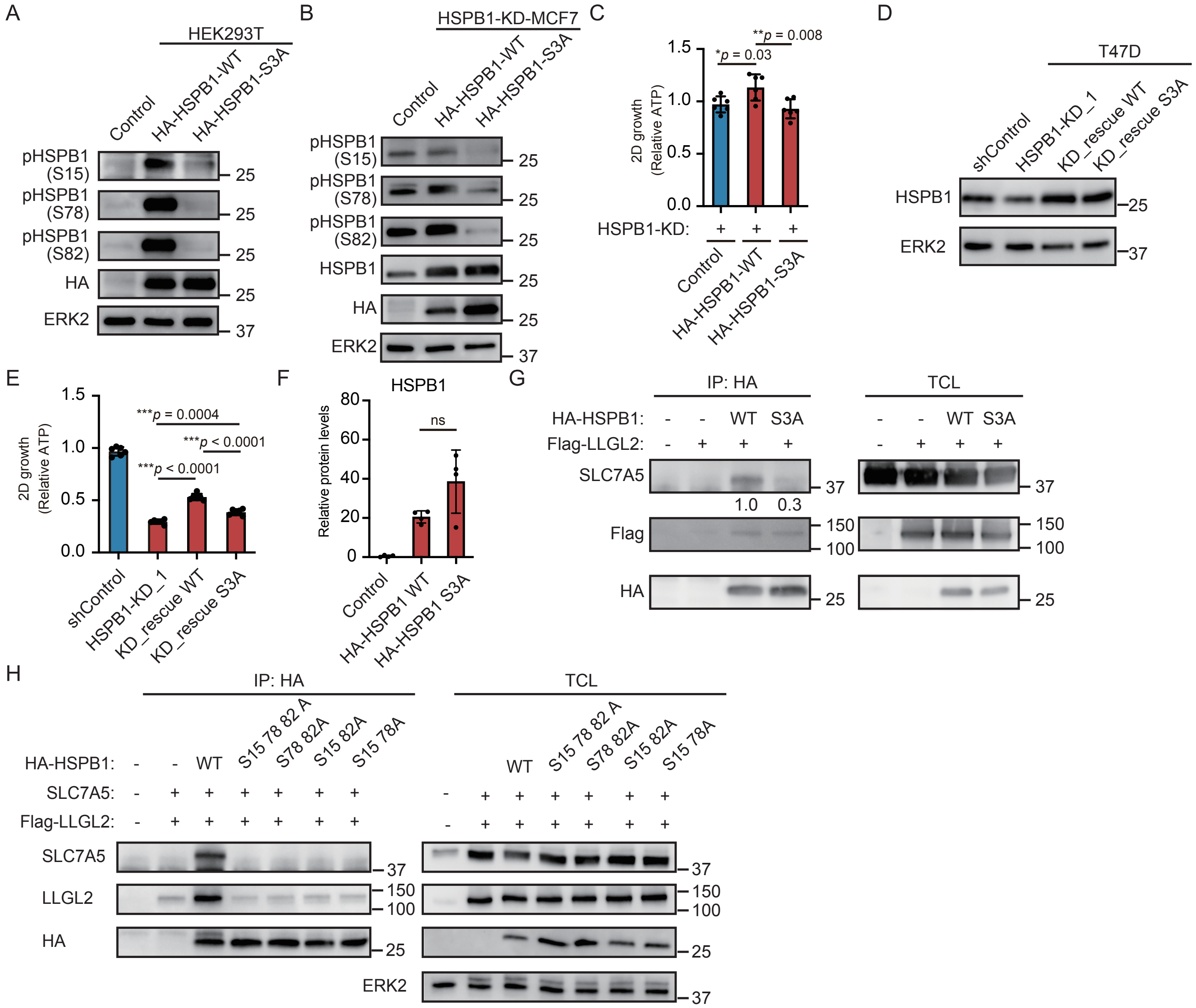

Supplementary Figure 3. Phosphorylated HSPB1 is required for the LLGL2-SLC7A5 interaction.

A, Antibody validation for phospho-HSPB1 at Ser 15, Ser 78, or Ser 82 using HA-HSPB1-WT, HA-HSPB1-S3A-expressing wild-type HEK293T cell lysates. **B**, The phosphorylation levels of shRNA-resistant HA-HSPB1-WT and shRNA-resistant HA-HSPB1-S3A in HSPB1-KD MCF-7 cells. **C**, Relative cell number of shRNA-resistant HA-HSPB1-WT- or HA-HSPB1-S3A-expressing HSPB1-KD MCF-7 cells by measuring intracellular ATP amount. **D**, HSPB1 expression in wild-type T47D cells. **E**, Relative cell number of HSPB1-KD, HSPB1-KD rescued T47D cells by measuring intracellular ATP amount. T47D cells were co-infected with the lentivirus that induces HSPB1-shRNA with or without shRNA-resistant wild-type HSPB1 or HSPB1-S3A mutant. Cells were spread in a 96-well plate, and the ATP amount was measured at Day 4. **F**, Relative protein levels of HA-HSPB1-WT and HA-HSPB1-S3A detected in mass spectrometry analysis in Fig. 3D. **G**, HSPB1-SLC7A5 interaction in wild-type HEK293T cells. HA-HSPB1-WT or HA-HSPB1-S3A was expressed with or without Flag-tagged LLGL2 in wild-type HEK293T cells and the cell lysates were immunoprecipitated with anti-HA antibody. The precipitants were immunoblotted with the indicated antibodies. **H**, The phospho-resistant HSPB1 mutants do not bind with SLC7A5 and LLGL2. The wild-type or phosphor-resistant mutants were expressed with SLC7A5 and Flag-LLGL2 in HSPB1-KO HEK293T cells. The lysates were immunoprecipitated with anti-HA antibody and the precipitants were immunoblotted with the indicated antibodies. Data **C, E,** and **F** are shown as mean ± s.d.; **C, E,** n=6, **F**, n=4. Statistical analysis performed by one-way ANOVA followed by Tukey's post-test (**C, E, F**).
