# Supplementary material for: The cellular stress sensor HSPB1 regulates the membrane localization of amino acid transporter SLC7A5 in breast cancer
Source: J Biol Chem. 2026 May 27;302(7):113197. doi: 10.1016/j.jbc.2026.113197 (PMC13311823; doi:10.1016/j.jbc.2026.113197)
Supplement: Supplementary Figure S4 [file mmc4.pdf]

Supplementary Fig. 4

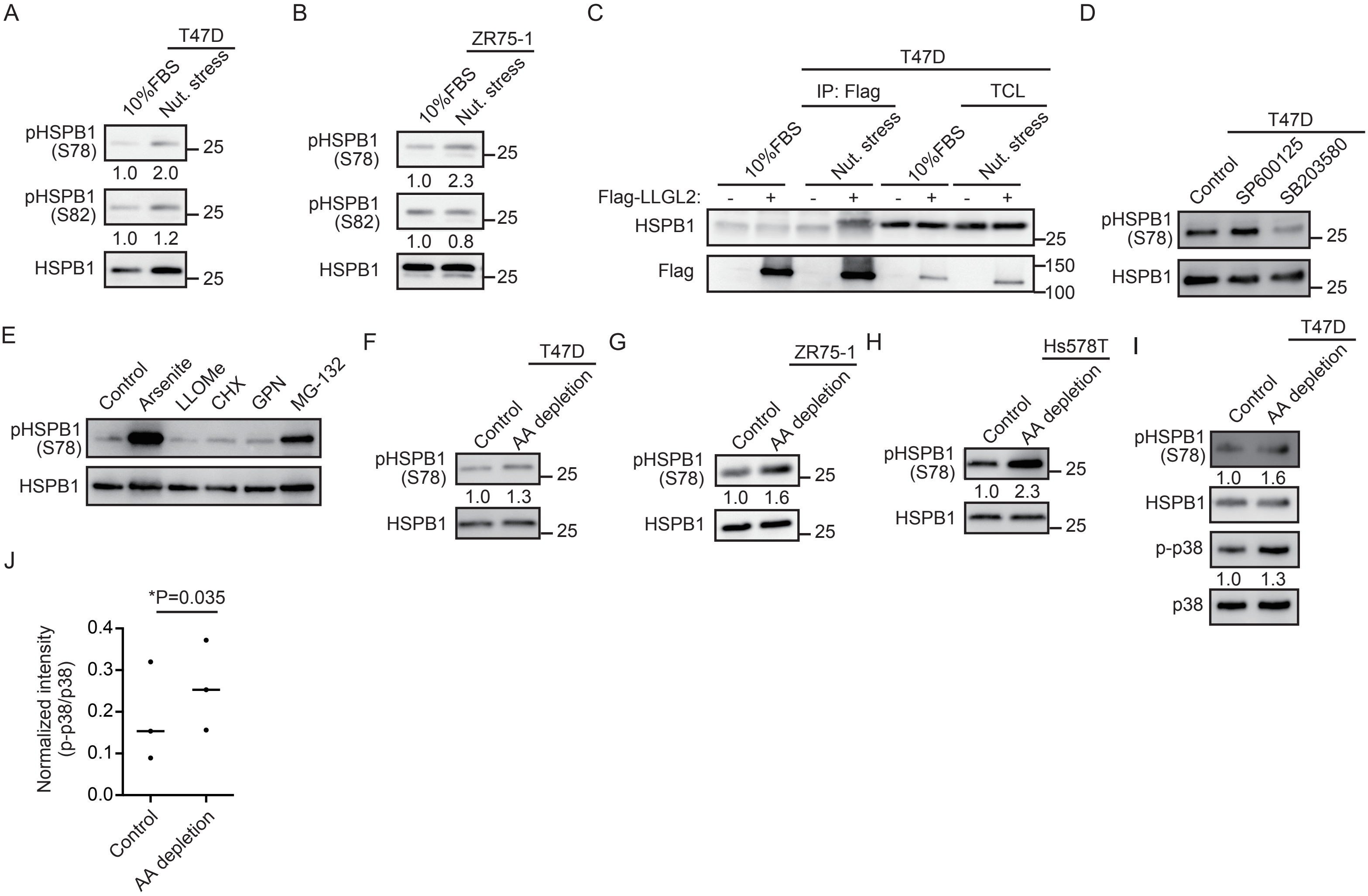

Supplementary Figure 4. Nutrient stress upregulates HSPB1 phosphorylation at Ser 78 in ER+ breast cancer.

A, The phosphorylation levels of HSPB1 in T47D under different culture conditions for two days. **B**, The phosphorylation levels of HSPB1 in ZR75-1 under different culture conditions for two days. **C**, HSPB1-LLGL2 interaction in T47D cells under different culture conditions. Cells were cultured in RPMI-1640 medium supplemented with 10% FBS and insulin or nutrient stress medium for two days. The cell lysates were immunoprecipitated with anti-Flag antibody and the precipitants were immunoblotted with the indicated antibodies. **D**, Stress MAPK inhibitors treatment in T47D cells. The phopho-HSPB1 at Ser 78 residue was examined by immunoblotting. **E**, Cellular stresses induce the phosphorylation of HSPB1 at Ser 78 in T47D cells. Each compound was treated with 100 μM at the final concentration. **F**, T47D cells were cultured in amino acid-free RPMI medium for 1 hour. The phospho-HSPB1 levels were assessed by immunoblotting. **G**, ZR75-1 cells were cultured in amino acid-free RPMI medium for 1 hour and the phopho-HSPB1 levels were examined by immunoblotting. **H**, Hs578T cells were cultured in amino acid-free RPMI medium and the phospho-HSPB1 levels were examined by immunoblot. **I**, T47D cells were cultured in AA-free RPMI medium and the phospho-HSPB1 and phosphor-p38 were examined by immunoblot. **J**, Normalized intensity of phospho-p38 in amino acids-depleted T47D cells. Data J is shown as mean with plots. J; n=3. Statistical analysis was conducted by two-tailed t-test.
