# Supplementary material for: The cellular stress sensor HSPB1 regulates the membrane localization of amino acid transporter SLC7A5 in breast cancer
Source: J Biol Chem. 2026 May 27;302(7):113197. doi: 10.1016/j.jbc.2026.113197 (PMC13311823; doi:10.1016/j.jbc.2026.113197)
Supplement: Supplementary Figure S5 [file mmc5.pdf]

Supplementary Fig. 5

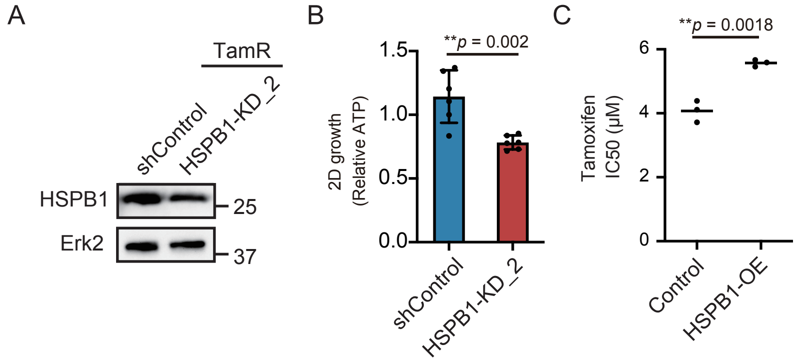

Supplementary Figure 5. HSPB1 overexpression induces tamoxifen resistance in ER+ breast cancer cells.

A, Immunoblot images of HSPB1-KD TamR MCF-7 cells. **B**, Relative cell number of HSPB1-KD TamR MCF-7 cells by measuring intracellular ATP amount. **C**, IC50 values of tamoxifen -treated HSPB1-OE MCF-7 cells. Data **B** and **C** are shown as mean ± s.d.; **B**, n=6, **C**, n=3. Statistical analysis was conducted by two-tailed Student's t-test (**B, C**).
